# The effects of Direct Payments on the well-being of older adults in England

**DOI:** 10.1007/s10433-025-00882-w

**Published:** 2025-08-22

**Authors:** Jinbao Zhang, Julien Forder

**Affiliations:** Personal Social Services Research Unit, University of Kent, Canterbury, UK

**Keywords:** Self-direction, Participant-directed care, Personal budgets, Cash for care, Person-centered care

## Abstract

Self-directed care is a model that allows older adults with disabilities to arrange home- and community-based services flexibly. This study examines the impact of England’s self-directed care—Direct Payments—on older adults’ well-being and explores how the effects vary among individual characteristics. This study used data from the English Longitudinal Study of Ageing (ELSA, 2012–2023) on adults aged 65 and older who used Direct Payments and managed care (*N* = 568). We used propensity score matching methods to estimate the impact of Direct Payments on unmet needs, depressive symptoms, and quality of life. Direct Payments reduced unmet needs and had non-significant effects on depressive symptoms and quality of life. The beneficial effects of Direct Payments were more salient among older adults who were younger, had experienced less physical loss, and had reported met needs in the preceding survey. Our findings suggest that Direct Payments effectively improve older adults’ care outcomes by addressing their unmet needs, with individuals possessing greater health capital being more likely to benefit from using them. Policymakers and practitioners should support the disadvantaged and mitigate disparities in well-being.

**Supplementary Information:**

The online version contains supplementary material available at 10.1007/s10433-025-00882-w.

## Introduction

Self-directed care has been widely adopted internationally to improve service quality (Carlson et al. 2007; Fleming et al. 2019). By giving community-dwelling individuals choice and control over their care arrangements, self-directed care schemes are presumed to be more responsive to individual preferences and needs than conventional “one-size-fits-all” service models (henceforth termed managed services). Extensive research has demonstrated the positive impact of self-directed care on well-being, especially for younger adults (Glendinning et al. 2008; Pattyn et al. 2021; Yoshino CA and Atkins 2023).

Concerns have been raised that older adults may not derive as much benefit from self-directed care as do younger adults and other groups (Carey et al. 2019; Damant et al. 2020). For example, self-directed care in England enhanced the quality of life of individuals with mental illness and increased satisfaction with services for younger physically impaired individuals (Glendinning et al. 2008). However, older adults aged 65 and over who used self-directed care reported lower psychological well-being (Glendinning et al. 2008; Netten et al. 2012; Moran et al. 2013). Similarly, Woolham and colleagues (2017) found that older adults (aged 75 +) who used self-directed care and managed services exhibited no statistically significant differences in their health status and social care-related quality of life. Therefore, more research is needed to investigate the effects of self-directed care among older adults, which is crucial for informing policymakers on how to promote self-direction and tailoring services to individual preferences and needs.

Additionally, few studies have explored the heterogeneous effects of self-directed care on older adults’ well-being. Individuals with different characteristics may not benefit equally from using self-directed care. While prior research has suggested that the advantaged group, typically younger, more affluent individuals with greater family support, are more inclined to use self-directed care (Leece and Leece 2006; Manthorpe et al. 2011), little is known about whether more or less advantaged older persons experience different outcomes from self-directed care.

To address these gaps, this study examines the impact of England’s self-directed care on well-being (i.e., unmet needs, depressive symptoms, and quality of life) among older people, using a nationally representative dataset and propensity score matching methods. Direct Payments are a crucial self-directed care program that older adults can utilize to purchase and manage their services flexibly, allowing them to receive personalized support (further details are discussed in the literature review section). Additionally, we explore the heterogeneous effects of Direct Payments utilization across individual characteristics. Our findings contribute to understanding the effectiveness of self-directed care for older adults. Also, the findings indicate who is less likely to benefit from using the service, offering crucial insights for local authorities to implement person-centered care and develop strategies for reducing inequality in well-being.

## Literature review

### Self-directed care and well-being

Self-determination theory is valuable for understanding how self-directed care, acting as an environmental factor, influences individual well-being. The theory posits that three universal psychological needs—autonomy (i.e., perceived choice and control over one’s life), relatedness (i.e., feeling connected to others), and competence (i.e., the ability to effectively interact with environments)—are essential for optimal functioning and well-being (Deci and Ryan 2012). The fulfillment of these needs is facilitated or constrained by social environments, including care policies and interpersonal relationships. As a crucial environmental factor, self-directed care policies shape self-determination through enabling individuals to organize service arrangements aligning with their preferences and needs, such as receiving care from trusted family members (DeCarlo et al. 2018; Pattyn et al. 2023). A large body of literature highlights the positive impacts of self-directed care (compared to managed services) on well-being, including delayed institutionalization, reduced acute care admissions and emergency department visits, as well as improved self-determination, sense of security, and quality of life (Benjamin and Matthias 2000; Carlson et al. 2007; Fleming et al. 2019; Pattyn et al. 2021, 2023; Yuan et al. 2022; Yoshino CA and Atkins 2023).

Despite growing evidence on the positive outcomes of self-directed care, concerns have been raised about the effectiveness of self-directed care for older adults due to their limited competence in regard to interacting with care systems (Williams et al. 2017; FitzGerald Murphy and Kelly 2019; Kelly et al. 2021). These concerns are particularly salient in England, where older adults have been found not to derive as much benefit from using self-directed care as younger individuals (Glendinning et al. 2008; Netten et al. 2012). For example, Moran and colleagues (2013) revealed that older adults in England using self-directed care reported lower self-rated health status and psychological well-being than those receiving managed services. These adverse outcomes were attributed to the burdens of organizing care arrangements and managing budgets, such as anxiety about misuse of budgets on inappropriate goods and over- or under-spending the budget. Similarly, Woolham and colleagues (2017) observed no significant difference in stress, self-rated health, or social care-related quality of life between Direct Payments users (aged 75 +) and managed services users. They suggest that this may reflect older adults’ limited capacity to exercise self-direction.

Given the mixed evidence on the effectiveness of self-directed care, more research is needed to examine its impact on older adults’ well-being. While empirical studies have focused on psychological well-being indicators, such as stress, self-rated health (e.g., EQ-5D-3L), and social care-related quality of life (e.g., ASCOT), little research has explored the impact of self-directed care on older adults’ unmet needs, primarily due to the lack of data specific to this population (Fleming et al. 2019; Davey 2021). Unmet needs are crucial for evaluating the effectiveness of public social care support (Vlachantoni 2019). Higher unmet needs are associated with elevated anxiety and depression, increased risk of hospital admissions and readmissions, and heightened medical spending (Huang et al. 2024). Factors such as younger age, being male, living alone, possessing fewer financial resources, experiencing more functional impairments, and having limited informal support are related to having more unmet needs (Dunatchik et al. 2019; Spiers et al. 2022; Vlachantoni et al. 2024; Patterson and Freedman 2025). Additionally, the lack of public social care services leads to unmet needs (Calderón-Jaramillo and Zueras 2023), yet there is little evidence on the role of different care policies (e.g., self-directed care vs. managed services). Therefore, investigating the impact of self-directed care on older adults’ unmet needs is essential to assess the effectiveness of social care, improve individual well-being, and reduce avoidable healthcare utilization and costs.

### Capital, administrative burden, and self-directed care

In self-directed approaches, administrative burdens shift from home care professionals and agencies to service users, potentially leading to unequal benefit among older adults (Woolham et al. 2017; Xie et al. 2024). Administrative burdens can manifest in various forms, including learning costs (e.g., gathering information about programs, eligibility criteria, and regulations on hiring care workers), compliance costs (e.g., spending budgets appropriately and reporting to governments), and psychological costs (e.g., perceived loss of autonomy when using formal care and stigma arising from means-tested programs). Managing these responsibilities requires individuals to possess the necessary capital and skills, which are disproportionately distributed (Halling and Baekgaard 2024; Bell et al. 2024). Bourdieu’s (1989) capital theory provides a valuable framework for conceptualizing these inequalities. He categorized unequally distributed, structurally based resources into three types—social, cultural, and economic capital—which shaped individuals’ ability to manage the administrative burdens and benefit from using self-directed care (Carey et al. 2019). Social capital involves resources embedded within individuals’ social networks. The presence of family members and social support could compensate for functional loss in older adults and help them exercise self-direction (Pattyn et al. 2023; Xie et al. 2024). Thus, social capital serves as a protective factor, helping older adults manage administrative burdens and enabling them to benefit from using self-directed care.

Cultural capital is defined as individuals’ symbolic and informational resources for action (Bourdieu 1989). A person’s education level is often used as an indicator representing cultural capital. First, education level is positively associated with perceived self-efficacy in managing administrative responsibilities. Educated individuals tend to be more confident in their ability to communicate with governments and receive positive responses to their demands (Gilad and Assouline 2024). Second, navigating care systems requires individuals to possess various knowledge and skills, such as information searching, financial literacy, and digital literacy (Xie et al. 2024). These forms of cultural capital are typically stronger among more educated individuals (Carey et al. 2019). Therefore, less educated individuals may find administrative burdens challenging to manage and be less likely to benefit from using self-directed care.

Economic capital refers to material wealth, principally income. Previous studies have emphasized pro-rich inequalities in formal care utilization within systems where personal budgets are distributed evenly (e.g., fixed amounts) across lower- and higher-income groups (Bakx et al. 2015; Albertini and Pavolini 2017; Lera et al. 2021). Nonetheless, evidence on the relationship between income and the ability to benefit from using self-directed care is limited (Carey et al. 2019). Florid and colleagues (2024) suggested that income did not moderate the association between formal care receipt and outcomes like depressive symptoms and quality of life. This occurred because formal care receipt represented a response to lower physical functioning rather than socio-economic gradients. Thus, older adults across income levels may derive equal benefit from using self-directed care, especially when personal budgets are allocated based on assessed care needs rather than flat rate payments.

In addition to these three types of capital, health capital relates to health-related resources, such as physical and mental capacity (Hyry-Honka et al. 2012). These resources are crucial for older adults to manage administrative burdens and benefit from using self-directed care (Pattyn et al. 2021). A large body of research has evaluated the effectiveness of self-directed care compared to managed services specifically among individuals with mental illnesses. These studies indicate that self-directed care users experience fewer unmet needs and higher service satisfaction, particularly when support brokers are available (Glendinning et al. 2008; Shen et al. 2008; Cook et al. 2023). While these studies focus on advocating self-direction among individuals with mental illnesses, less is known about whether service users benefit equally from using self-directed care. Using interview data (*N* = 81) from the England’s Individual Budgets pilot program, Davey (2021) found that older adults with lower dependency levels were more likely to benefit from using self-directed care. Overall, although self-directed care yields positive outcomes for individuals with limited health capital, these benefits may be disproportionally distributed among them.

### Direct Payments in England

While self-directed care programs vary considerably across countries, they are essentially mechanisms whereby service users are empowered to direct their own care arrangements. A cross-national comparative study of 11 self-directed programs across seven countries, including England, Australia, Germany, and the USA, identified two widely adopted policy features: enabling older adults to flexibly transition between self-directed care and managed services, and providing information to access services (Zhang et al. 2023). Among these programs, England’s Direct Payments ranked third in consumer direction. Given this relatively high performance, analyzing Direct Payments can provide insights into the effectiveness of self-directed care and benefit other countries developing similar policies.

In England, local authorities are responsible for providing social care for older adults. Services are means tested, primarily targeting those experiencing functional impairments and possessing income and assets below the statutory threshold. Eligible individuals can receive a wide range of services, including long-term support, short-term support (to maximize independence), and other services, such as end-of-life care and ongoing low-level support. Older adults living in the community are entitled to personal budgets covering their assessed care costs. Personal budgets can be managed in three ways: (1) the local authority manages the budget and arranges the services, (2) a third party (e.g., care providers) holds the budget and organizes the services, or (3) users receive Direct Payments and direct services themselves. The first two options constitute managed services. Regardless of whether older adults receive managed services or Direct Payments, local authorities are required to allocate sufficient personal budgets based on eligible care needs and consider individualized reasonable preferences for service arrangements.

Direct Payments users are able to employ relatives and friends as care workers, which is an appealing option for older adults who prefer family caregiving. However, co-residing family members are ineligible for employment through this program. Additionally, Direct Payments can be spent creatively on care-related goods and services, such as attending singing classes, playing cards with old friends, and going to the pub. Local authorities also permit older adults to receive a mix of managed services and Direct Payments as they wish.

To mitigate administrative burdens and support self-direction, the government has implemented several policy initiatives. First, older adults with limited mental capacity can designate family members as representatives to manage Direct Payments. In some cases, professionals, including general practitioners, social workers, and Independent Mental Capacity Advocate, can be nominated as representatives (Age UK 2024), which is crucial for older adults without family support. Second, local authorities are required to offer information and support to promote self-direction (National Audit Office 2016). Through this provision, older adults can access information about their rights, entitlements, and service options. They are also meaningfully involved in their care planning, which enables them to make informed choices about how their care is funded and delivered. Lastly, pre-paid cards have been introduced to facilitate real-time auditing of spending by local authorities through electronic platforms. While being criticized for limiting service flexibility, such as prohibiting cash withdrawals, this approach reduces the need for older adults to report budget spending (Davey 2021). These initiatives are expected to mitigate administrative burdens and facilitate self-direction, especially for older adults with limited health, social, and cultural capital (Pattyn et al. 2023; Xie et al. 2024).

## The present study

Drawing on the preceding literature review, this study examines the impact of Direct Payments on older adults’ well-being and explores the heterogeneous effects across individual characteristics. Given the policy improvements supporting self-direction, we formulate the following hypotheses:

### H1a

Older adults using Direct Payments experience less unmet needs than those receiving managed services.

### H1b

Older adults using Direct Payments report fewer depressive symptoms than those receiving managed services.

### H1c

Older adults using Direct Payments have a higher quality of life than those receiving managed services.

### H2a

The beneficial effects of using Direct Payments are equally distributed across older adults with different levels of health capital because of the policy efforts to support self-direction.

### H2b

The beneficial effects of using Direct Payments are equally distributed across older adults with different levels of economic capital, as personal budgets are allocated based on care needs rather than flat rate payments.

### H2c

The beneficial effects of using Direct Payments are equally distributed across older adults with different levels of social capital, reflecting policy efforts to support self-direction.

### H2d

The beneficial effects of using Direct Payments are equally distributed across older adults with different levels of cultural capital because of the policy efforts to support self-direction.

## Methods

### Data

This study used data from the English Longitudinal Study of Ageing (ELSA), a nationally representative study of community-dwelling adults aged 50 and above (NatCen Social Research et al. 2023). The baseline survey took place in 2002–2003, and follow-ups were conducted biennially. ELSA gathers respondents’ information on sociodemographic and health characteristics, physical and social environments, and access to social care. This study used data from waves 6 to 10 (2012–2023) because, for the first time, wave 6 included the social care module and detailed information on access to Direct Payments. The final analytic sample comprised 458 participants aged 65 and over across five waves, totaling 568 observations. This included 128 managed services users (166 observations) and 330 Direct Payments users (402 observations). The sample selection process is visualized in Supplementary Figure S-1.

### Measures

#### Dependent variables

Well-being was measured by unmet needs, depressive symptoms, and quality of life. We assessed unmet needs based on participants’ responses to questions on whether they reported difficulties in performing six activities of daily living (ADLs, including eating, dressing, bathing, using the toilet, walking across the room, and getting in and out of bed) and received help for the respective need. If participants reported a need but did not receive help (regardless of source: formal or informal care) for this activity, we coded the item as an unmet need. By adding up the number of unmet needs, we created a continuous variable for absolute unmet ADLs needs, ranging from 0 (no unmet needs) to 6 (needs not met at all). Additionally, we created a dichotomous variable to indicate whether participants had unmet ADLs needs (0 = met, 1 = unmet). Depressive symptoms were assessed using the eight-item Centre for Epidemiologic Studies Depression Scale (CES-D8). Participants were asked whether they had felt or behaved in a certain way during the past week, such as “felt sad” and “felt lonely.” All eight items were summed to a variable that captured a general score for depressive symptoms. Higher scores indicate more depressive symptoms. Quality of life was measured based on the CASP-19 instrument. Example questions include “How often do you feel that your life has meaning?” and “How often do you feel that age prevents you from doing things you like.” Participants were asked to indicate the extent to which each of the 19 items described their feelings about life using a four-point scale (0 = never, 1 = not often, 2 = sometimes, and 3 = often). The scale scores were summed to create the measure of quality of life, with a high score indicating good quality of life.

#### Independent variable

Direct Payments utilization was a dichotomous variable (0 = managed services, 1 = Direct Payments). ELSA asks participants to report how local authorities arrange payment for their care services, including Direct Payments and money managed by local authorities, councils, or social services organizations. Notably, participants are allowed to combine Direct Payments and managed services, which is referred to as Part Direct Payment; these people were categorized as Direct Payments users in this study (*N* = 13).

#### Control variables

Health capital was measured with age, functional capabilities, and unmet needs reported in the preceding survey. Age was measured in years. Functional capabilities were assessed using the number of limitations with six ADLs. Detailed items can be found in the measure on unmet needs. A higher number of limitations mean lower functional capabilities. Additionally, a dichotomous variable was created to indicate whether respondents experienced any difficulties performing nine instrumental activities of daily living (IADLs), such as grocery shopping, taking medications, managing money, and working around the house and garden.

Social capital was assessed using household size and the receipt of informal care. The receipt of informal care was measured by whether respondents received instrumental support with ADLs and IADLs from relatives (e.g., spouses and adult children) in the last month (0 = no, 1 = yes). Economic capital was measured by annual income, a financial-derived variable from the ELSA dataset. It was equivalized using the modified OECD equivalence scale to adjust for the size and composition of a household and logarithmically transformed to obtain a more normal distribution. Cultural capital was measured by whether participants had obtained a formal educational qualification (0 = no, 1 = yes).

### Statistical analysis

An ideal approach to answering our research questions would have been to randomly assign community-dwelling older people needing social care to a treatment group (Direct Payments users) and a control group (managed services users) and compare the well-being of the two groups. However, randomly assigning older people to a treatment and control group is unethical because this may undermine individuals’ autonomy in choosing the option that best suits their needs and preferences. Studies using observational data face the challenge of selection bias, as users of Direct Payments and managed services differ in many characteristics that may substantially affect individual well-being. Accordingly, the results are biased by confounding factors. To address this issue, we used a propensity score matching (PSM) method that balanced covariates between individuals and formed a treatment group and a comparable control group. PSM creates quasi-randomization to reduce selection biases, making it a widely used approach for conducting causal inferences using observational data (Abadie and Imbens 2016).

We used PSM to estimate the difference between the average outcome for older people who used Direct Payments and those who received managed services (the average treatment effect on the treated, ATT). The estimation equation can be presented as:

$$\text{ATT}= E\left[Y(1)- Y(0)|DP=1\right]$$

where $Y(1)$ is the outcome if older people received Direct Payments, and $Y(0)$ indicates the outcome if older people used managed services. Since each respondent is observed only in either the treatment or the control group, either $Y(1)$ or $Y(0)$ is observed for each individual, and the other must be estimated. Specifically, we conducted PSM in three steps to solve selection bias and estimate ATT. First, we selected control variables and calculated propensity scores. Variables that were associated with individual well-being were included because including variables unrelated to the outcome but highly associated with the treatment assignment increased the variance in the sample outcome and bias in estimating treatment effects (Stuart 2010; Kainz et al. 2017). Based on previous studies (Dunatchik et al. 2019; Spiers et al. 2022; Vlachantoni et al. 2024; Patterson and Freedman 2025), we included nine predictors (sex, one lagged dependent variable, and seven control variables described in the control variables section) that were related to well-being. The longitudinal structure of the ELSA dataset allowed us to employ the preceding waves to define the covariates and the following waves to define the treatment status. For example, lagged covariates in wave 6 were derived from wave 5. This strategy offers two advantages: 1) avoiding the bias caused by the treatment assignment influencing control variables and 2) including lagged dependent variables to estimate propensity scores, thus controlling for time-invariant unobservable confounders (Balbo and Arpino 2016; de Zwart et al. 2017). Using a multivariate logistic regression that included the eight predictors elicited, we calculated the propensity scores, i.e., the conditional probability of older people receiving Direct Payments.

**Fig. 1:**
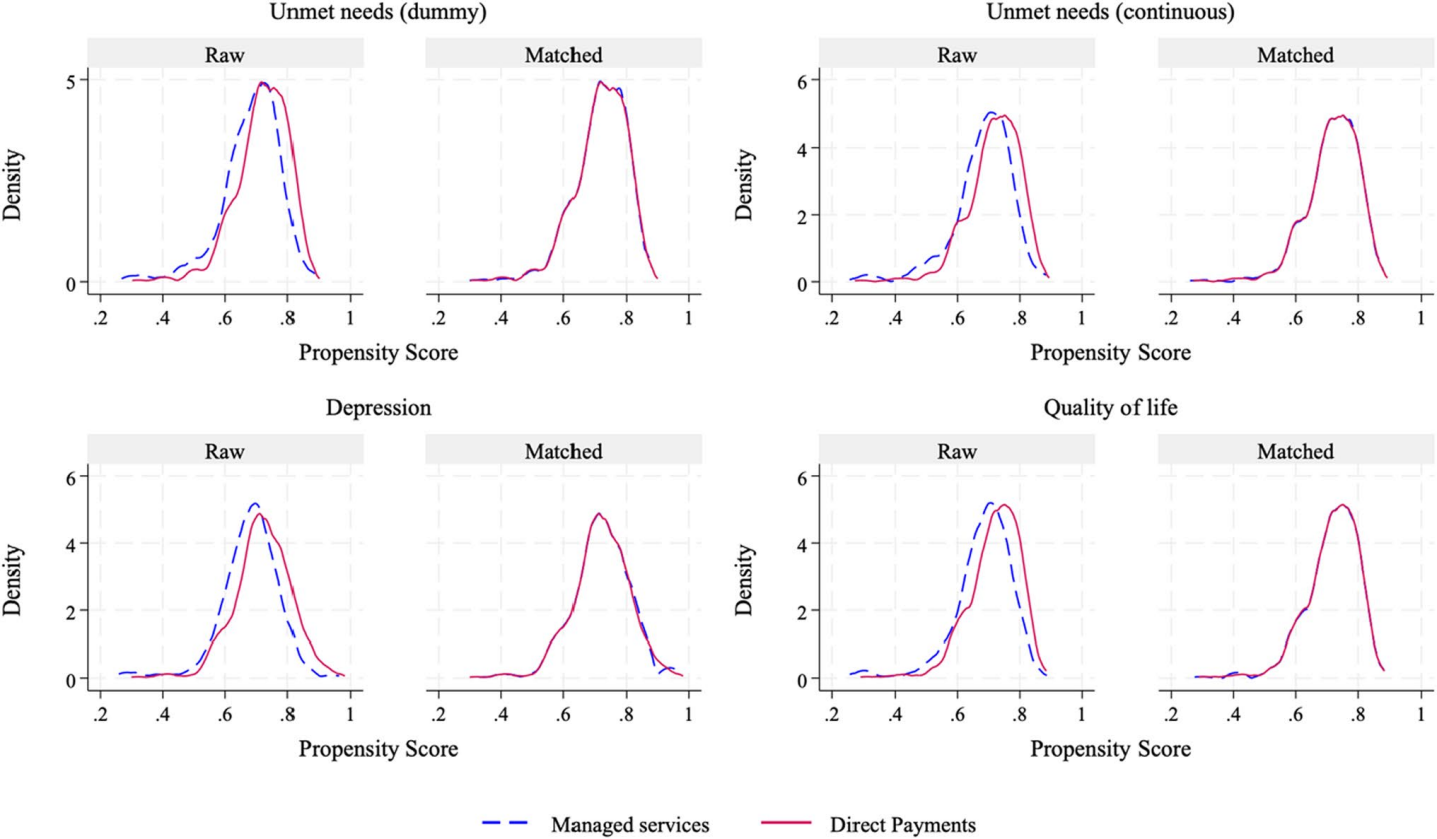
Distribution of propensity scores before and after matching

Second, we assessed the quality of the matches using three strategies: (1) plotting the area of common support, (2) calculating the standardized bias to examine the balance of each control variable, and (3) reporting Rubin’s B, which reflects the absolute standardized difference in the means of the propensity scores in the treatment and control groups. Common support refers to the overlap in the distribution of propensity scores between the treatment and control groups. Figure 1 illustrates the distribution of the propensity scores for the well-being of older adults before and after matching (common support). After matching, the distributions of the propensity scores between the treatment and control groups overlapped, which indicates a strong balance in the propensity score and satisfies the common support assumption.

**Fig. 2:**
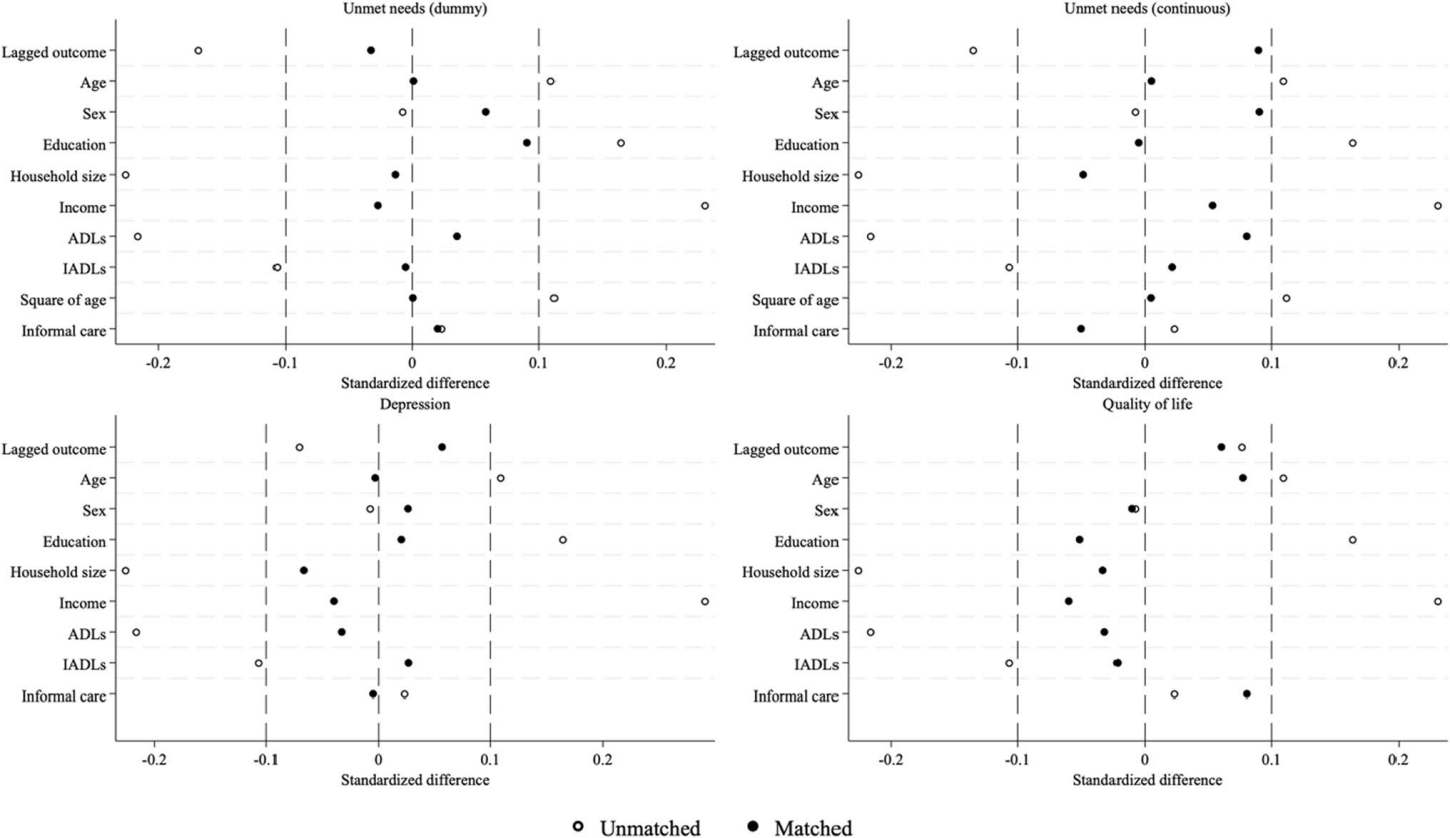
Standardized bias across control variables

Figure 2 shows the standardized bias of each control variable before and after matching. Standardized bias compares the difference in group means and divides it by the standard deviation. Our matching strategies substantially reduced the standardized bias for all variables, with the values for all baseline characteristics falling below the threshold (< 0.1) after matching, indicating adequate balance. Additionally, we calculated Rubin’s B for each model; a value of < 25% indicates balanced treatment and control groups (Stuart 2010). The sample exhibited imbalance before matching, with Rubin’s B ranging from 41.8 to 45.7%, but achieved balance after matching, with Rubin’s B ranging from 12.5 to 17.5%. Overall, our method achieves a better balance after matching.

Third, after creating a balanced propensity score, we used nearest neighbor matching (1:1) with replacement to pair Direct Payments users with managed services users. Matching with replacement can produce reliable treatment effects estimates (Bottigliengo et al. 2021). To reduce bias from observable confounders, we adopted a caliper with 0.20 of the standard deviation of the logit of the propensity score (Austin 2011).

To ensure the robustness of our results, we report two types of ATT: (1) ATT and robust standard errors based on Abadie and Imbens’ (2016) approach, where the estimation approach considers the fact that the propensity score is estimated rather than known; (2) doubly robust estimates that used regression analysis and adjusted for all control variables for the matched sample using the weights from our matching method as probability weights, which corrected for the small residual difference in the distribution of the control variables (Nguyen et al. 2017; de Zwart et al. 2017). In addition, all of the regression models were estimated with cluster-robust standard errors. Missing data were handled by listwise deletion.

In addition to assessing the ATT of Direct Payments on well-being, we also examined the variation in treatment effects across individuals (i.e., treatment effect heterogeneity) using a matching-smoothing (MS) method. The MS approach uses the interaction between the treatment assignment and the propensity for treatment to address selection bias (Xie et al. 2012; Guo and Fraser 2015). As a nonparametric method, the MS method does not impose a functional form on effect heterogeneity, which helps researchers flexibly uncover patterns of treatment effects heterogeneity over the range of propensity scores.

## Results

### Descriptive statistics

Table 1 presents the descriptive statistics of the sample before propensity score matching. The sample sizes for Direct Payments and managed services users were 402 and 166, respectively. Compared to older people who received managed services, those using Direct Payments were more likely to have a smaller household size, possess a higher annual income, experience fewer limitations in ADLs, and report fewer unmet needs.

**Table 1:** Descriptive statistics before propensity score matching (*N* = 568)

Variable	All (*N* = 568)	Managed services (*N* = 166)	Direct Payments (*N* = 402)	*P*
	Mean (SD)	Mean (SD)	Mean (SD)	
*Dependent variables*				
Unmet needs (dummy)	0.62 (0.49)	0.79 (0.41)	0.55 (0.50)	***
Unmet needs (continuous)	2.09 (2.13)	2.77 (2.10)	1.80 (2.08)	***
Depression	2.24 (2.18)	2.08 (2.15)	2.31 (2.19)	
Quality of life	18.55 (17.05)	16.39 (16.61)	19.44 (17.17)	
*Control variables*^a^				
Age	78.91 (7.99)	78.30 (7.92)	79.17 (8.02)	
Sex	0.35 (0.48)	0.35 (0.48)	0.35 (0.48)	
Education	0.58 (0.49)	0.52 (0.50)	0.60 (0.49)	
Household size	1.58 (0.87)	1.73 (1.05)	1.52 (0.78)	**
Income	301.40 (162.91)	270.30 (119.93)	314.24 (176.19)	**
ADLs	1.74 (1.88)	2.04 (1.98)	1.62 (1.82)	*
IADLs	0.70 (0.46)	0.73 (0.44)	0.69 (0.46)	
Informal care	0.44 (0.50)	0.43 (0.50)	0.45 (0.50)	
Unmet needs (dummy)	0.31 (0.46)	0.37 (0.48)	0.29 (0.45)	
Unmet needs (continuous)	0.88 (1.61)	1.03 (1.66)	0.81 (1.58)	*
Depression	2.26 (2.17)	2.37 (2.27)	2.21 (2.13)	
Quality of life	24.44 (16.63)	23.54 (16.78)	24.81 (16.57)	

^a^This table reports lagged values for all control variables. *P < 0.05, **P < 0.01, ***P < 0.001

Logistic regression analyses were used to examine factors associated with Direct Payments use (see Supplementary Table S-1). The regression results largely reconfirm the descriptive findings that Direct Payments users reported higher annual income, fewer limitations in ADLs, and smaller household size than their counterparts using managed services. These findings align with prior research indicating that older adults from disadvantaged backgrounds, especially those with limited financial resources and higher care needs, were less likely to use self-directed care (Leece and Leece 2006; Carey et al. 2019; Malbon et al. 2022). The negative association between household size and Direct Payments utilization may reflect the institutional constraint that prohibits older adults from hiring co-residing family members as caregivers. Since older adults in larger households often rely on co-residing family support, they may find Direct Payments less appealing (due to hiring restrictions) and choose managed services instead. In contrast, those in smaller households—who have greater access to non-cohabiting family caregivers—may use Direct Payments to employ these individuals.

### Effects of Direct Payments on well-being

Table 2 presents the effects of Direct Payments on older adults’ well-being, including unmet needs, depression scores, and quality of life. Model 1 (the first column) shows the results based on Abadie and Imbens’s (2016) approach, and Model 2 (the second column) reports the doubly robust estimates. Using Direct Payments reduced the likelihood of experiencing unmet needs by 20% in both Model 1 (*P* < 0.01) and Model 2 (*P* < 0.001). Users of Direct Payments reported 0.64 (Model 1, *P* < 0.01) or 0.70 (Model 2, *P* < 0.01) fewer unmet needs compared to users of managed services. These results support Hypothesis 1a. Older people who used Direct Payments reported more depressive symptoms and higher quality of life than their counterparts. Yet, the differences were not statistically significant at the 5% level.

**Table 2:** Effects of Direct Payments on well-being (*N* = 568)

Dependent variables	(1) AI’s method			(2) Doubly robust estimates		
	ATT	SE	*P*	ATT	SE	*P*
Unmet needs (dummy)	−0.20	0.065	**	−0.20	0.050	***
Unmet needs (continuous)	−0.64	0.244	**	−0.70	0.232	**
Depression	0.44	0.239		0.37	0.220	
Quality of life	2.47	2.158		2.146	2.172	

*P < .0.05, **P < 0.01, ***P < 0.001

### Heterogeneous treatment effects

**Fig. 3:**
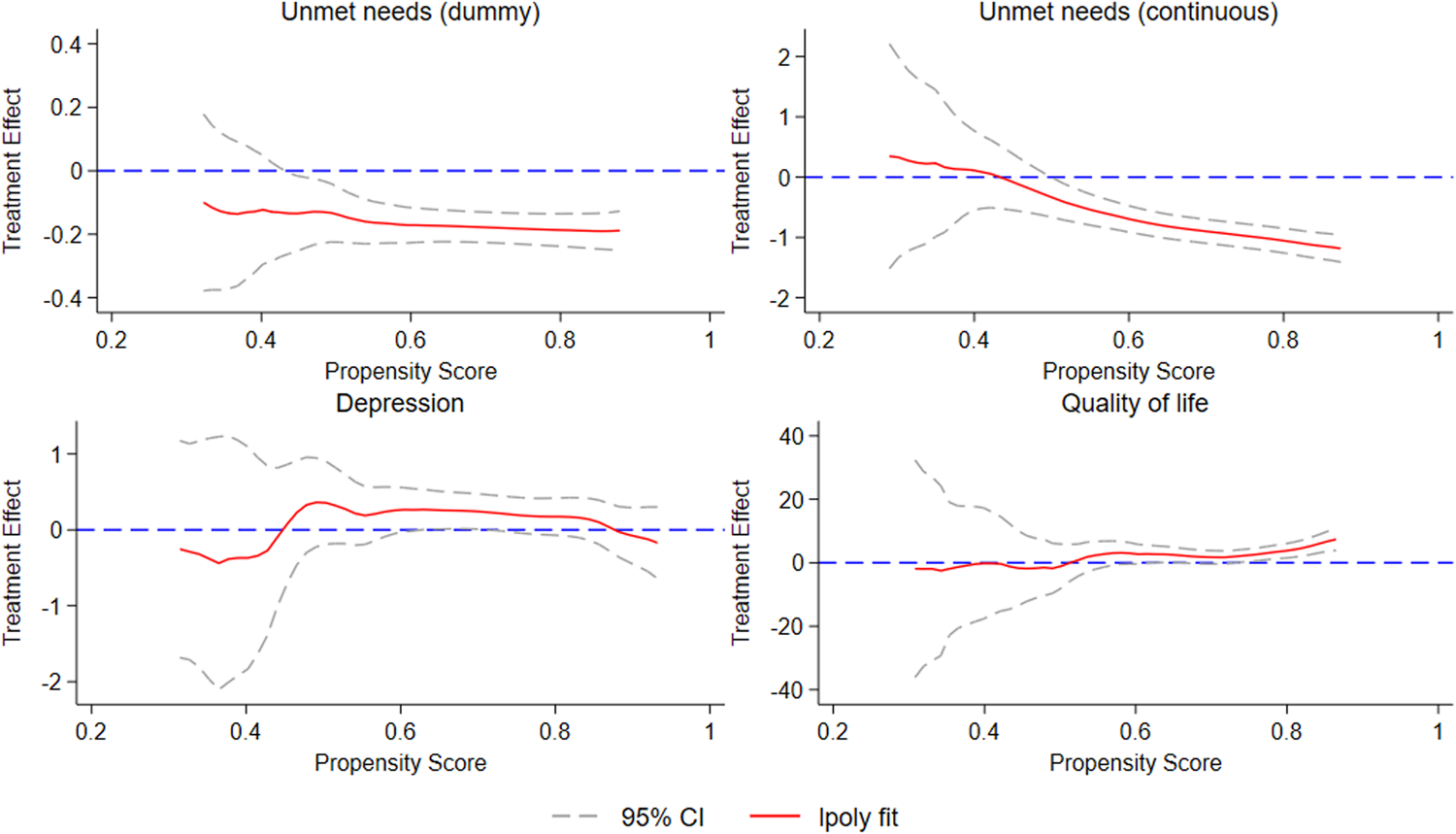
Heterogeneous treatment effects

We next investigated the heterogeneity in the effect of Direct Payments utilization on well-being based on the propensity for using Direct Payments. Figure 3 presents the results of local polynomial matching–smoothing treatment effect heterogeneity. The *x*-axis denotes the continuous propensity scores for using Direct Payments. The *y*-axis represents the observed differences between older people with Direct Payments and those with managed services on the four well-being variables: unmet needs (dummy and continuous variables), depression scores, and quality of life. The top panel of Fig. 3 shows how the estimated effects on unmet needs varied across propensity scores. Both subpanels (top left and right) indicate that the effects of Direct Payments on unmet needs were largest for older adults with a higher probability of using Direct Payments. For depressive symptoms (bottom left panel) and quality of life (bottom right panel), treatment effects remained close to zero and showed little variation across propensity scores, except for a small but significant effect on quality of life at the highest propensity scores. These results suggest little heterogeneity in treatment effects for the two outcomes. While these results are useful to reveal whether heterogeneity in treatment effects exists across propensity scores, examining heterogeneity by individual characteristics offers greater policy relevance—as we explore next.

**Fig. 4:**
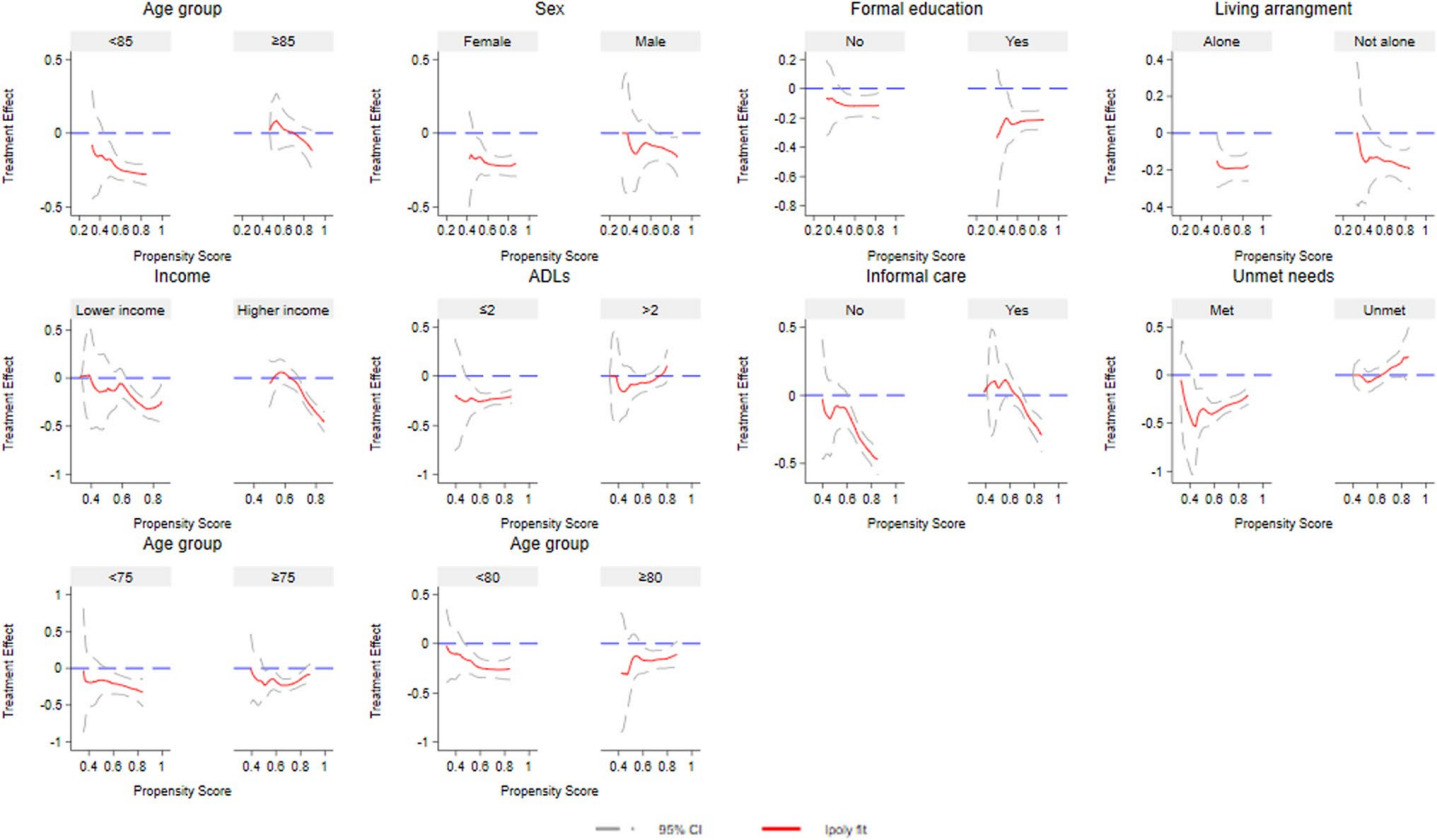
Heterogeneous treatment effects of Direct Payments on unmet needs (dummy). Income was stratified into lower and higher quantiles

**Fig. 5:**
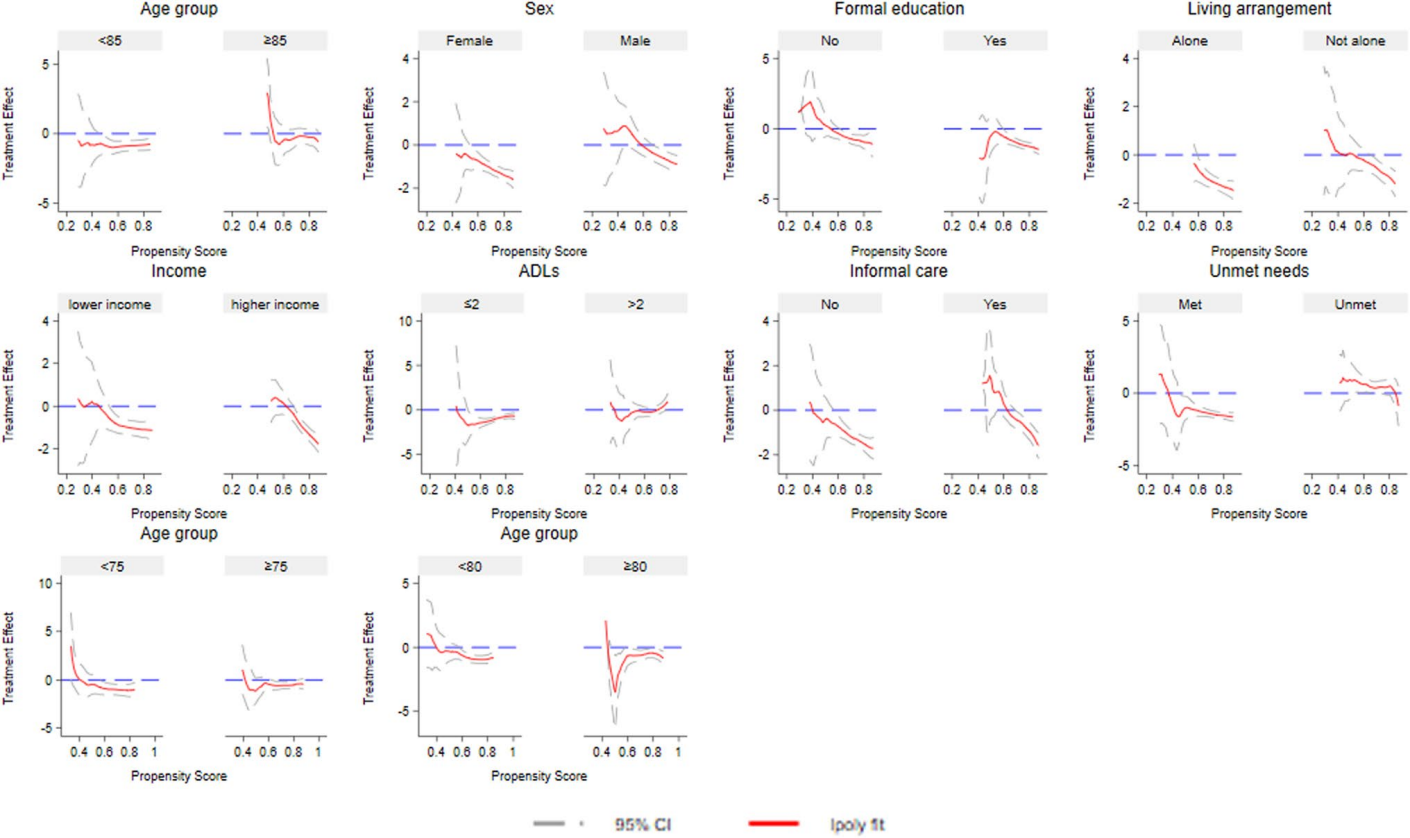
Heterogeneous treatment effects of Direct Payments on unmet needs (continuous). Income was stratified into lower and higher quantiles

Given the significant effects of Direct Payments on unmet needs, we examined the treatment effect heterogeneity by group based on individual characteristics. The heterogeneous treatment effects on the dummy unmet needs variable (Fig. 4) exhibit similar patterns to those observed for the continuous unmet needs variable (Fig. 5), supporting the robustness of our results. First, Direct Payments significantly reduced both the likelihood and the number of unmet needs for older adults under 85, with fewer (≤ 2) limitations in ADLs and experiencing no unmet needs in their last interview, particularly when these individuals were more inclined to use Direct Payments. For example, the negative effect of Direct Payments on unmet needs became statistically significant for individuals aged below 85 with higher propensity scores, whereas no significant effect was observed for those aged 85 and over. Given the arbitrary choice of age groups, we tested alternative cutoffs (i.e., 75 and 80). Results showed that the beneficial effects of Direct Payments were statistically significant for younger older adults aged below 75 or 80 and having higher propensity scores. While Direct Payments reduced the likelihood of experiencing unmet needs (Fig. 4) for those with more advanced age (75 + or 80 +), these effects were statistically significant at the 5% level only for a narrow range of propensity scores. One possible explanation is that the beneficial effects of Direct Payments decrease with age. Individuals aged 75 to 85 may still benefit from using Direct Payments, with statistically significant results observed for those aged 75 (or 80) and over within a small range of propensity scores. Overall, the results reject Hypothesis 2a.

Additionally, we did not find significant heterogeneous treatment effects among other group characteristics, including sex, income, educational qualifications, living arrangements, and the availability of informal care. For example, the effects of Direct Payments on unmet needs were negative and statistically significant at the 5% level among individuals with moderate-to-high propensity scores, regardless of formal education status. This indicates no evidence of heterogeneity in effects by education level.

### Sensitivity analysis

To assess the robustness of our results, we conducted two types of sensitivity analysis. First, given that the ELSA employs repeated measures to track individuals over time, we performed random effects and population-averaged effects models. As shown in Supplementary Table S-2, Direct Payments had significant adverse impacts on unmet needs (both dummy and continuous variables) while showing non-significantly positive effects on depression scores and quality of life. These results coincide with our main analysis.

Second, we performed the Rosenbaum bound analysis to examine the influence of hidden bias due to unobservable confounders on our results. The propensity score matching method assumes that an older person’s decision to use Direct Payments solely depends on observable factors. This assumption may be violated because factors such as preferences for self-direction in service arrangements can influence the service decision. The bound analysis examined whether an inference about treatment effects may be sensitive to unobservable confounders, and the result is presented in Supplementary Table S-3. The treatment effects of Direct Payments were robust to hidden bias when the significance level of the Γ values was less than 0.05 and sensitive if the Γ values changed from statistically significant to non-significant. In the benchmark scenario (Γ = 1.0), the results were statistically significant for unmet needs, which aligned with our main findings. Though the associations of depressive symptoms and quality of life with Direct Payments were significant at Γ=1.0, both were the most sensitive to hidden bias among the four well-being variables, as they became non-significant at Γ=1.3 and 1.1, respectively. The result for unmet needs (dummy) was the most robust to the presence of hidden bias, and the result became non-significant until Γ=2.3. However, the result for unmet needs (continuous) was much less robust because the result became non-significant when Γ=1.5.

## Discussion

This study investigated the impact of Direct Payments on older adults’ well-being and explored whether and how heterogeneous effects existed within this population. Using the ELSA dataset and propensity score matching methods, we found that Direct Payments significantly improved care outcomes by reducing unmet needs, with advantaged groups showing a greater inclination to benefit from using it. Our findings highlight the effectiveness of Direct Payments and the underlying heterogeneity among older adults. These insights could help governments develop strategies for implementing self-directed care schemes and supporting individuals who gain less from Direct Payments.

Older people who used Direct Payments were less likely to experience unmet needs and had fewer unmet needs than those receiving managed services, suggesting that Direct Payments enhance older adults’ care outcomes. When interpreting this finding, limitations in measuring unmet needs must be acknowledged. The ELSA data may underestimate the extent of unmet needs because met needs were operationalized solely as the receipt of support for a specific task. In reality, receiving help does not guarantee that needs are fully met, as older adults may perceive the assistance they receive as inadequate or unsatisfying. Like prior studies (Vlachantoni 2019; Vlachantoni et al. 2024), our measures capture absolute care gaps rather than suboptimal service provision—the former often requiring more urgent policy interventions. While previous studies on Direct Payments in England have primarily focused on the well-being of older people, this study is one of the first to examine the impact of Direct Payments on unmet needs—a policy priority and an indicator of the effectiveness of social care. Our results align with the evidence from the USA, indicating that self-directed care effectively decreases unmet needs among older people (Benjamin and Matthias 2000; Carlson et al. 2007). This may reflect local authorities’ initiatives to alleviate administrative burdens and facilitate self-direction, thereby addressing barriers identified in earlier research (Moran et al. 2013; Woolham et al. 2017).

However, Direct Payments showed no statistically significant effects on depressive symptoms or quality of life. This aligns with Woolham and colleagues’ (2017) findings regarding comparable psychological well-being between older adults using Direct Payments and those receiving managed services. One interpretation is that both groups receive limited personal budgets for addressing psychological needs (Moran et al. 2013), potentially explaining these null results. Future research could investigate the mechanisms underlying these outcomes.

Our analysis reveals a heterogeneous treatment effect of Direct Payments on unmet needs among older adults with different levels of health capital. Older adults who were younger, had fewer physical impairments, and whose needs had been met in the preceding survey were more likely to benefit from using Direct Payments than their counterparts. This finding echoes previous studies that emphasize health status as critical in exercising self-direction (Williams et al. 2017; Carey et al. 2019; Davey 2021; Pattyn et al. 2021). For example, older adults with severe health conditions are unable to leave home for leisure activities over extended periods, although this flexibility is permitted by Direct Payments (Damant et al. 2020). However, we observed no heterogeneity across economic, social, and cultural capital. This may be attributed to policy efforts that mitigate administrative burdens, enabling those with varying capital to achieve equal benefits.

### Implications for policy and research

This study contributes to the current literature in two ways. First, it advances research on the effectiveness of Direct Payments and self-directed care for older people. We used unmet personal care needs as one of our outcome variables, an aspect that is less explored in the existing literature (Fleming et al. 2019). Additionally, we provide up-to-date evidence on Direct Payments, considering local authorities’ initiatives to mitigate administrative burdens and facilitate self-direction. Second, instead of assuming that older adults benefit equally from using Direct Payments, this study highlights that Direct Payments favor the advantaged, particularly those with more health capital. These findings enrich the literature on self-directed care and inequality in well-being by providing more nuanced evidence.

The findings of this study also have implications for practice, particularly in regard to how policymakers and practitioners implement self-directed care. Our analysis suggests that older people in England benefit from using Direct Payments. Therefore, local authorities could promote access to it and increase its take-up rate, especially when aiming to reduce absolute care gaps among older people. Additionally, individuals with more health capital are more prone to benefiting from using Direct Payments. This highlights the importance of practitioners and the government offering support for the disadvantaged, particularly those of an older age, with more physical impairments and prior unmet needs, to mitigate disparities in well-being.

### Limitations

This study acknowledges the following limitations. First, our analyses rest on the unverifiable assumption of ignorability, meaning that after adjusting for a set of observable characteristics, the treatment assignments are independent of outcome variables. Due to the limited data, we could not include all factors, such as care relationships and the number of care providers in local communities, which may explain the association found. Second, this study used observational data, and the unobserved time-varying covariates could not be adjusted for, potentially resulting in biased estimates. However, we included lagged dependent variables as one of the variables to address the selection bias resulting from the unobservable time-constant confounding factors. Third, although we used data from a nationally representative dataset—ELSA—our study participants were recruited from various regions across England. Local authorities have implemented different regulations regarding Direct Payments, such as budget amounts and whether individuals can withdraw money from their budgets, which may also affect well-being. Due to the small sample size, our analysis could not capture these policy variations across regions. Lastly, the findings on heterogeneous treatment effects should be interpreted cautiously due to the small sample size, which may limit generalizability. Future research with a larger sample size is needed to validate these subgroup differences. Despite these limitations, this study provides evidence supporting the implementation of Direct Payments among older people and identifies heterogeneous effects of Direct Payments within this population.

## Supplementary Information

Below is the link to the electronic supplementary material.

Supplementary file1 (DOCX 54 KB)

## Data Availability

The ELSA data are freely available and can be accessed through the UK Data Service: 10.5255/UKDA-SN-5050-29. Statistical analyses used in the study are available from the first author upon request.
