# Supplementary material for: The effects of Direct Payments on the well-being of older adults in England
Source: Eur J Ageing. 2025 Aug 22;22(1):45. doi: 10.1007/s10433-025-00882-w (PMC12373561; doi:10.1007/s10433-025-00882-w)
Supplement: Supplementary file 1 — Supplementary file1 (DOCX 54 KB) [file 10433_2025_882_MOESM1_ESM.docx]

**Supplementary Materials**

**Table S-1**

*Factors associated with the use of Direct Payments (N = 568)*

|  | Model 1 | Model 2 |
| --- | --- | --- |
| Age | 0.010 (0.012) | 0.009 (0.012) |
| Sex | -0.037 (0.203) | -0.076 (0.206) |
| Education | 0.242 (0.201) | 0.275 (0.203) |
| Household size | -0.220* (0.105) | -0.248* (0.109) |
| Income | 0.451* (0.178) | 0.473** (0.181) |
| ADLs | -0.108^+^ (0.058) | -0.112^+^ (0.068) |
| IADLs | 0.009 (0.242) | 0.009 (0.242) |
| Informal care | 0.123 (0.200) | 0.119 (0.202) |
| Unmet needs |  | 0.007 (0.072) |
| Depression |  | -0.050 (0.045) |
| Quality of life |  | -0.003 (0.006) |
| Pseudo-*R*^2^ | 0.029 | 0.031 |

*Notes*: All models adjusted for lagged covariates and reported robust standard errors. ^+^ *P* < .1, **P* < .05, ***P* < .01, ****P* < .001.

**Table S-2**

*Random effects and population-averaged models (N = 568)*

|  | Random effects model | | Population-averaged model | |
| --- | --- | --- | --- | --- |
|  | *b* | *SE* | *b* | *SE* |
| Unmet needs (dummy) | -0.15*** | 0.039 | -0.17*** | 0.038 |
| Unmet needs (Continuous) | -0.73*** | 0.197 | -0.73*** | 0.167 |
| Depression | 0.27 | 0.188 | 0.28 | 0.173 |
| Quality of life | 2.16 | 1.449 | 2.26 | 1.414 |

*Notes*: *b* denotes coefficients, and *SE* represents standard errors. In the random effects model, we used cluster-robust standard errors. **P* < .05, ***P* < .01, ****P* < .001.

**Table S-3**

*Rosenbaum-bound sensitivity analysis*

|  | 1) Unmet needs (dummy) | | 2) Unmet needs (continuous) | | 3) Depression | | 4) Quality of life | |
| --- | --- | --- | --- | --- | --- | --- | --- | --- |
| Γ | Sig+ | Sig- | Sig+ | Sig- | Sig+ | Sig- | Sig+ | Sig- |
| 1.0 | 0.000 | 0.000 | 0.000 | 0.000 | 0.000 | 0.000 | 0.011 | 0.011 |
| 1.1 | 0.000 | 0.000 | 0.000 | 0.000 | 0.005 | 0.000 | 0.071 | 0.001 |
| 1.2 | 0.000 | 0.000 | 0.000 | 0.001 | 0.033 | 0.000 | 0.231 | 0.000 |
| 1.3 | 0.000 | 0.000 | 0.000 | 0.007 | 0.120 | 0.000 | 0.475 | 0.000 |
| 1.4 | 0.000 | 0.000 | 0.000 | 0.031 | 0.287 | 0.000 | 0.712 | 0.000 |
| 1.5 | 0.000 | 0.000 | 0.000 | 0.095 | 0.503 | 0.000 | 0.873 | 0.000 |
| 1.6 | 0.000 | 0.000 | 0.000 | 0.212 | 0.706 | 0.000 | 0.954 | 0.000 |
| 1.7 | 0.000 | 0.001 | 0.000 | 0.376 | 0.852 | 0.000 | 0.986 | 0.000 |
| 1.8 | 0.000 | 0.002 | 0.000 | 0.555 | 0.935 | 0.000 | 0.996 | 0.000 |
| 1.9 | 0.000 | 0.004 | 0.000 | 0.715 | 0.975 | 0.000 | 0.999 | 0.000 |
| 2.0 | 0.000 | 0.009 | 0.000 | 0.836 | 0.992 | 0.000 | 1.000 | 0.000 |
| 2.1 | 0.000 | 0.019 | 0.000 | 0.914 | 0.997 | 0.000 | 1.000 | 0.000 |
| 2.2 | 0.000 | 0.034 | 0.000 | 0.959 | 0.999 | 0.000 | 1.000 | 0.000 |
| 2.3 | 0.000 | 0.057 | 0.000 | 0.982 | 1.000 | 0.000 | 1.000 | 0.000 |

*Notes*: Γ: log odds of differential assignment due to unobserved factors. Sig+: upper bound significance level. Sig-: lower bound significance level.


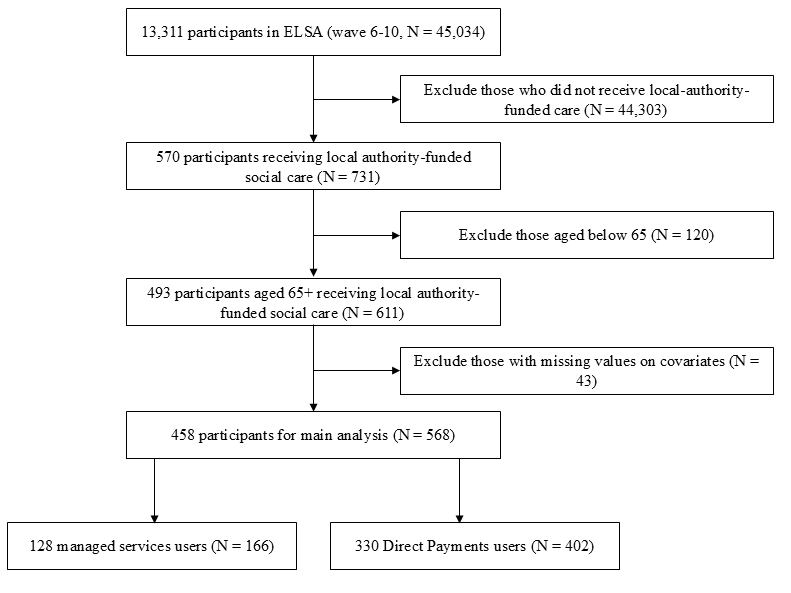


**Figure S-1**

*Flowchart of sample selection from the ELSA*
